# Barriers and drivers to COVID-19 vaccination among the migrant and non-migrant population in Germany, 2021

**DOI:** 10.1093/eurpub/ckae017

**Published:** 2024-02-09

**Authors:** Elisa Wulkotte, Nora Schmid-Küpke, Kayvan Bozorgmehr, Oliver Razum, Ole Wichmann, Julia Neufeind

**Affiliations:** Immunization Unit, Robert Koch Institute, Berlin, Germany; Immunization Unit, Robert Koch Institute, Berlin, Germany; School of Public Health, Bielefeld University, Bielefeld, Germany; Section Equity Studies & Migration, University Hospital Heidelberg, Heidelberg, Germany; School of Public Health, Bielefeld University, Bielefeld, Germany; Immunization Unit, Robert Koch Institute, Berlin, Germany; Immunization Unit, Robert Koch Institute, Berlin, Germany

## Abstract

**Background:**

During the Coronavirus Disease 2019 (COVID-19) pandemic, immunization programmes struggled to reach all population groups equally. While migrant groups face multiple barriers to health systems, including vaccination, little is known about their vaccine uptake.

**Methods:**

We conducted a cross-sectional telephone survey on adults with and without migration history in Germany to investigate barriers and drivers to COVID-19 vaccination (11 April 2021 to 18 December 2021). Interviews were conducted in six languages. We used logistic regression models and a mediation model to analyze the association between migration history and vaccine uptake. Furthermore, we determined the effect of psychological determinants (5C model) on vaccine uptake.

**Results:**

The survey comprised 2039 individuals, including 1015 with migration history. Of these, 448 were interviews conducted in languages other than German. Individuals with migration history had a significantly lower vaccine uptake but, while still unvaccinated, had a higher intention to get vaccinated (*P* = 0.015) compared with those without migration history. The association between migration history and vaccine uptake was no longer significant when other factors were included in the regression model (odds ratio = 0.9; 95% confidence interval: 0.57–1.47). Socio-economic index, language skills and discrimination experience fully mediated this association. Among the psychological determinants, ‘higher confidence’ and ‘higher collective responsibility’ increased the chance of individuals with migration history to be vaccinated.

**Conclusion:**

Migration history alone cannot explain vaccine uptake; socio-economic index, language skills and discrimination experiences need to be considered. To achieve vaccine equity, future public health policy should aim to reduce relevant barriers through tailored interventions.

## Introduction

The Coronavirus Disease 2019 (COVID-19) pandemic posed multiple challenges to governments worldwide, including how to make vaccines accessible to a large population as quickly as possible and how to achieve high vaccination coverage. By late December 2020, when the COVID-19 vaccine became available in Germany, the capacity of immunization services and vaccine quantities were limited. Due to high demand in the general population,^1^ the vaccine was initially prioritized for population groups at high risk.^2^ With more vaccines becoming available in mid-2021, most people willing to get the vaccine received it. In late summer of 2021, the vaccine supply was higher than demand in the population, bringing the vaccination roll-out to a halt.^3^^,^^4^ At this point, vaccination coverage was 70.1% for 18- to 59-year-olds and 84.3% for those aged 60+ (data as of 30 September 2021).^4^ Germany aimed to reach a vaccination coverage of at least 85% for those aged 12–59 and 90% for those aged 60+.^5^ As these targets were not reached, there was a heightened interest in better characterizing the unvaccinated population and identifying enablers and barriers to COVID-19 vaccine uptake.

Migrant populations were at higher risk for a SARS-CoV-2 infection^6^^,^^7^ and had a higher COVID-19 mortality.^6^ This can be explained by social factors such as living and working conditions.^6^^,^^7^ It is, therefore, desirable to also achieve a high vaccination coverage in this group. However, previous surveys on COVID-19 vaccination demand have suggested that migrants might be one of several groups with low vaccine uptake in Germany.^8^^,^^9^ Today, people with a migration history make up more than one-quarter of the population living in Germany.^10^ Still, migrant groups have to overcome multiple barriers to healthcare systems, leading to a lower utilization of healthcare services. These include language barriers, legal regulations that restrict access to services, a lower socio-economic status and the experience of discrimination,^11–15^ all of which might be valid barrier to vaccine uptake as well.^16^ These barriers could be augmented by a lack of knowledge about vaccination, worries about vaccine safety and distrust in healthcare systems, which was also found to be more prominent among migrant populations in Europe.^16^ Barriers and drivers to COVID-19 vaccination, in particular, have been examined in only a few studies, focussing on migrant populations in Europe,^16–18^ while in Germany, very little research has been conducted so far to fully understand COVID-19 uptake among migrants.

To fill this research gap and to provide insights for tailoring the COVID-19 immunization programme, we assessed COVID-19 vaccine uptake and intention among the migrant and non-migrant population groups in Germany, as well as possible barriers and drivers. We addressed the following research questions:

Q1: Do COVID-19 vaccine uptake and vaccination intention differ between individuals with and without migration history?

Q2: Which characteristics explain possible differences in vaccine uptake between the populations with and without migration history?

Q3: Are psychological determinants associated with COVID-19 vaccine uptake, and is this association modified by migration history?

## Methods

### Survey design and population

Data were collected as part of the COVIMO study, a repeated cross-sectional telephone survey of the German adult population on COVID-19 vaccine demand. Data collection for the above-mentioned research questions took place in November and December 2021, i.e. nearly one year into the vaccination roll-out.

For our analysis, we aimed to recruit approximately 1000 participants with and 1000 without migration history. Individuals with migration history have been regarded as those who have either immigrated themselves to Germany or who have at least one parent who was not born in Germany. Interviews were offered in one of six languages frequently spoken in Germany^10^: German, Arabic, Turkish, Russian, Polish and English. People who immigrated themselves and people interviewed in languages other than German were oversampled to increase the statistical power. To realize the planned sample size, we had two separate samples: Sample A was representative of the general population of Germany (75% without and 25% with migration history). Sample B comprised individuals with migration history in order to numerically boost this group. The sampling procedure and participant groups are described in Supplementary figure S1.

Participants were recruited by a market and social research institute on behalf of the Robert Koch Institute, using randomly generated mobile and landline numbers and interviewed by computer-assisted telephone interview. To identify individuals who do not speak German, specific language codes were used. The randomly selected individuals were asked for their country of birth and countries of parents’ births to establish migration status.

The ethics commission of the Berlin Medical Chamber (#Eth-59/22) and the data protection officer at the Robert Koch Institute approved the survey. Participation in the study was voluntary. Participants were informed of the study’s aim and provided their oral consent before the interview started. No person-identifying data were collected during the interviews, and all data were treated confidentially to maintain participants’ anonymity.

### Measures

#### Vaccine measures

Participants were asked about the number of received COVID-19 vaccine doses. We built a binary variable vaccine uptake, indicating whether a person had been vaccinated at least once or was unvaccinated (coded as 1 and 0). Unvaccinated participants stated their intention to get vaccinated on a 5-point Likert scale from ‘definitely not vaccinate’ to ‘definitely vaccinate’ (coded 1–5).

We used the 5C model to assess a set of psychological determinants on vaccine uptake.^19^ The 5C is a validated and commonly used model to explain vaccination behaviour. It measures confidence (trust in vaccine and healthcare system), collective responsibility (perceive vaccination as a community measure to prevent the spread of the virus), constraints (perceived physical or individual barriers), calculation (extensive risk-benefit assessment) and complacency (no awareness of the risk of disease) with a set of validated questions.^19^ We used one item to assess collective responsibility, calculation and complacency. Considering that some migrant groups have to overcome multiple barriers in the healthcare system and might have trust issues, we implemented four constraints items (e.g. ‘It is difficult for me to get to the vaccination location’) and two confidence items (e.g. ‘I have complete trust in the safety of the COVID-19 vaccine’) into our questionnaire. In addition, we used two items derived from exploratory questions about reasons for (not) vaccinating from previous COVIMO survey waves^1^: ‘I will get vaccinated to regain my freedom’ (freedom); ‘I feel pressured to get vaccinated against COVID-19’ (pressure). The wording of the original 5C items was slightly adapted for telephone interviews, and all items were answered on a 5-point Likert scale from ‘does not apply at all’ to ‘applies completely’ (coded 1–5).

#### Socio-demographics and other characteristics

Socio-demographic data on age, sex, region (northern, eastern, southern and western Germany), mother tongue and country of birth (own and parents’) was obtained. Using information on the country of birth, we coded a binary variable of migration history (0: no; 1: yes). Education (1: low; 2: moderate; 3: high educational attainment) was captured by two questions about the highest education level completed and the highest professional qualification attained. The income indicator was monthly net equivalent household income, which is based on the net household income variable and was calculated following the Organisation for Economic Co-operation and Development equivalence scale.^20^ We operationalized socio-economic status by transforming education and income to a socio-economic index (range 2–14), according to Lampert et al.^21^

Participants assessed their German language skills on a 5-point Likert scale from ‘very poor’ to ‘very good’ (coded 1–5). We coded a new German language skills variable, adding information on the mother tongue as an additional category (6-point scale: 1: very poor self-assessed German language skills; 5: very good self-assessed German language skills; 6: German as mother tongue).

We assessed the frequency of discrimination experiences in the healthcare system, using an adapted version of the everyday discrimination scale.^22^^,^^23^ Participants indicated how often they were treated unfairly or worse than others in the healthcare sector (e.g. received worse service or were treated with less respect; 1: never, 5: very often).

All items used in the interviews are listed and explained in more detail here.

### Statistical analysis

Analyses were conducted using the statistical software STATA^24^ and R.^25^ Codes are provided online.

Descriptive statistics were calculated from weighted data. Weighting included design weights to adjust for the probability of accessibility of mobile and fixed network samples and adaptability weighting for sex, age, region, migration history and education. Inferential statistics were based on unweighted data.

For our first research question, we calculated the COVID-19 vaccination coverage —mean of the binary variable vaccine uptake, including 95% confidence intervals (CI)—for the migrant and non-migrant samples as well as for the participants with different levels of German language skills. We tested between-group differences with a χ^2^ test (significance level of 0.05). We further calculated the means of vaccination intention of unvaccinated participants and tested differences between participants with and without migration history via a *t*-test (significance level of 0.05). We report Cohen’s *d* as an estimate of the effect size.

To explain differences in vaccine uptake between groups with and without migration history (research question 2), we first developed a causal diagram [directed acyclic graph (DAG); see Supplementary figure S2). We performed binary logistic regressions with complete case analysis to examine the association between migration history and vaccine uptake, controlling for age, socio-economic index, discrimination experience and German language skills (stepwise regression). We report odds ratios (ORs) with 95% CIs as a measure of association and Mc Fadden’s *R*^2^ as a measure of model fit. To consider possible multicollinearity, we computed variance inflation factors (VIFs) and interpreted values <5 as presenting no multicollinearity issues.

To examine the effect of the socio-economic index, discrimination experience and German language skills on migration history and vaccine uptake, we performed a mediation analysis with the R package PROCESS.^26^ A parallel mediation model was applied with migration history as the independent variable, vaccine uptake as the dependent variable and socio-economic index, discrimination experience and German language skills as mediators. Age was added as a covariate to the model.

We further analyzed the relationship between the 5C psychological determinants and vaccine uptake stratified by migration history (research question 3). For this purpose, we built the mean score of the four constraints and the two confidence items, respectively. Two binary logistic regression models (complete case analysis) were established with the five 5C items and two additional items (pressure, freedom) as the independent variables and vaccine uptake as the dependent variable: one model for the migrant sample and another model for the non-migrant sample. The effects were controlled for age and socio-economic index. We report ORs with 95% CI and Mc Fadden’s *R*^2^. To test multicollinearity issues, we computed VIFs.

## Results

For sample A (a representative sample of the general population of Germany), 1285 interviews were completed; the response rate was 24.2% (calculation following AAPOR Combined Response Rate 3^27^). The number of completed interviews for sample B (boost for individuals with migration history) was 754. A response rate for sample B cannot be reported because there was no reference for the total number of individuals with migration history in the mobile and landline sample. In total, 2039 interviews were completed, including 1015 interviews with participants with migration history.

Among all participants, 51.1% were female, and the mean age was 52 years (SD* *=* *19). The study population is described in table 1.

**Table 1:** Description of the study population

	Participants with migration history		Participants without migration history	
	Weighted data	Non-weighted data	Weighted data	Non-weighted data
Age, years				
Mean (SD)	50.7 (19.2)	54.6 (17.7)	52.2 (18.6)	56.0 (16.6)
Sex, *n* (%)				
Male	263 (50.2)	477 (47.0)	734 (48.4)	504 (49.6)
Female	257 (49.8)	538 (53.0)	783 (51.6)	513 (50.4)
Region, *n* (%)				
Northern Federal States	86 (16.6)	178 (17.5)	282 (18.6)	175 (17.2)
Eastern Federal States	77 (14.9)	180 (17.7)	282 (18.6)	232 (22.8)
Southern Federal States	162 (31.3)	297 (29.3)	428 (28.2)	261 (25.7)
Western Federal States	193 (37.29)	360 (35.5)	524 (34.5)	348 (34.3)
Education, *n* (%)				
Low	140 (27.7)	147 (14.6)	142 (9.4)	44 (4.3)
Moderate	192 (37.9)	329 (32.6)	870 (57.8)	445 (44.0)
High	274 (34.4)	533 (52.8)	494 (32.8)	523 (51.7)
Monthly net equivalent income, €				
Mean (SD)	1,565.6 (1211.8)	1,782.3 (1268.2)	2,268.9 (1359.4)	2,525.7 (1440.3)
Socio-economic index (range, 2–14)				
Mean (SD)	6.9 (3.3)	8.1 (3.2)	8.5 (2.8)	9.6 (2.8)
Migration history, *n* (%)				
Immigrated themselves	411 (79.4)	792 (78.0)		
Direct descendants of immigrated parents	106 (20.6)	223 (22.0)		
Length of stay in Germany, years				
Mean (SD)	23.8 (17.1)	24.5 (15.5)		
Mother tongue, *n* (%)				
German	137 (26.7)	306 (30.3)		
Non-German	377 (73.3)	704 (69.7)		
Interview language, *n* (%)				
German	284 (54.8)	572 (56.4)		
Arabic	33 (6.4)	57 (5.6)		
English	23 (4.5)	47 (4.6)		
Polish	7 (1.4)	23 (2.3)		
Russian	79 (15.2)	211 (20.8)		
Turkish	91 (17.6)	105 (10.3)		

### COVID-19 vaccine uptake and vaccination intention

Of all participants, 90.3% (95% CI: 89.0–91.6) stated that they were vaccinated against COVID-19 at least once. Vaccination coverage (%) was calculated with weighted data, but we report unweighted absolute numbers (*n*).

**Figure 1:**
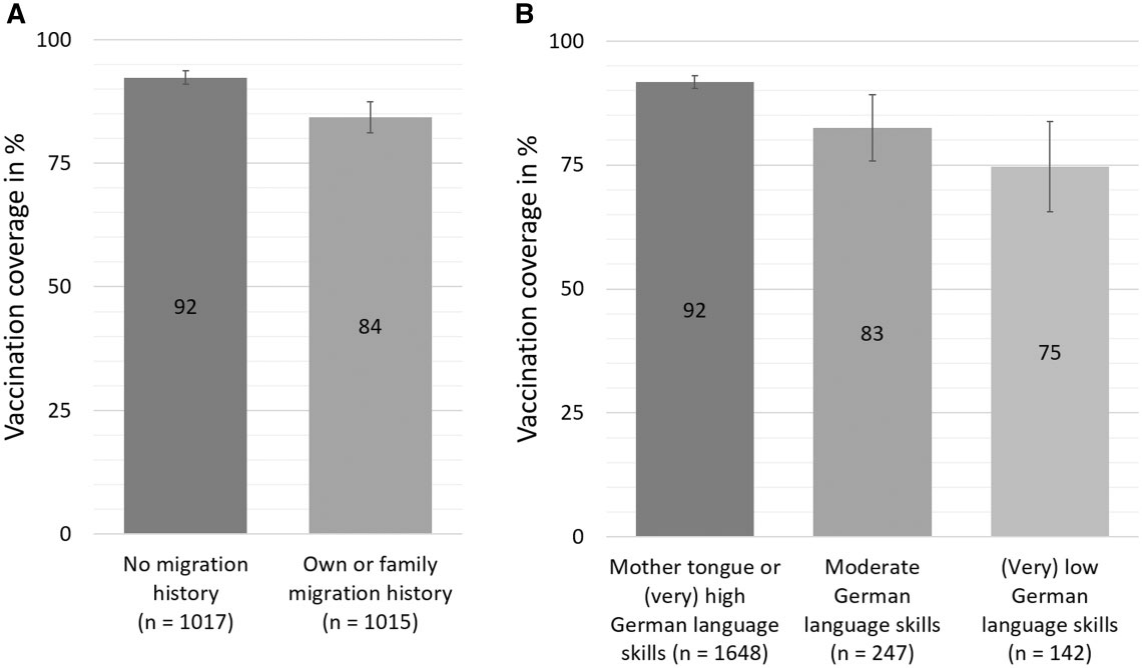
Vaccination coverage (vaccinated at least once) according to migration history (A) and German language skills (B)

Figure 1 shows that the estimated vaccination coverage was significantly higher among participants without as compared with those with migration history [χ^2^(1) = 28.07, *P *=* *0.000]. Participants with German as their mother tongue or self-rated (very) good German language skills had a significantly higher vaccine uptake than participants whose German language skills were moderate [χ^2^(1) = 11.5, *P *=* *0.002] and participants who rated their German language skills as (very) poor [χ^2^(1) = 21.14, *P *=* *0.000]. No significant difference in vaccination coverage was found between participants with moderate and participants with (very) low rated German language skills.

Of all unvaccinated participants (*n *=* *209), 48.6% said they were unlikely to get vaccinated or would not get vaccinated under any circumstances, 21.9% intended to get vaccinated, and 29.5% remained undecided about their vaccination decision. On the 5-point Likert scale, the vaccination intention was higher [*t*(207) = 2.46, *P *=* *0.015, *d *=* −*0.5] among unvaccinated participants of the group with (*M* = 3.0, SD = 1.6, *n *=* *137) compared with those of the group without migration history (*M* = 2.35, SD = 1.1, *n *=* *71).

### Migration history as a potential predictor of COVID-19 vaccine uptake

The initial regression model shows that participants with migration history were less likely to be vaccinated than those without migration history (model 1; *β *= 0.48, 95% CI: 0.36–0.65). The association between migration history and vaccine uptake became weaker when age, socio-economic index and discrimination experience were added to the model. By adding German language skills to the model, the OR of migration history was no longer significant (final model; *β *= 0.92, 95% CI: 0.57–1.47). In the final model, COVID-19 vaccine uptake was significantly associated with German language skills (*β *= 1.23, 95% CI: 1.05–1.45), discrimination experience (*β *= 0.77, 95% CI: 0.64–0.93), socio-economic index (*β *= 1.12, 95% CI: 1.04–1.18) and age (*β *= 1.02, 95% CI: 1.01–1.03). There was no multicollinearity in our models (VIFs < 5). All results from the multiple logistic regression models are reported in Supplementary table S2.

**Figure 2:**
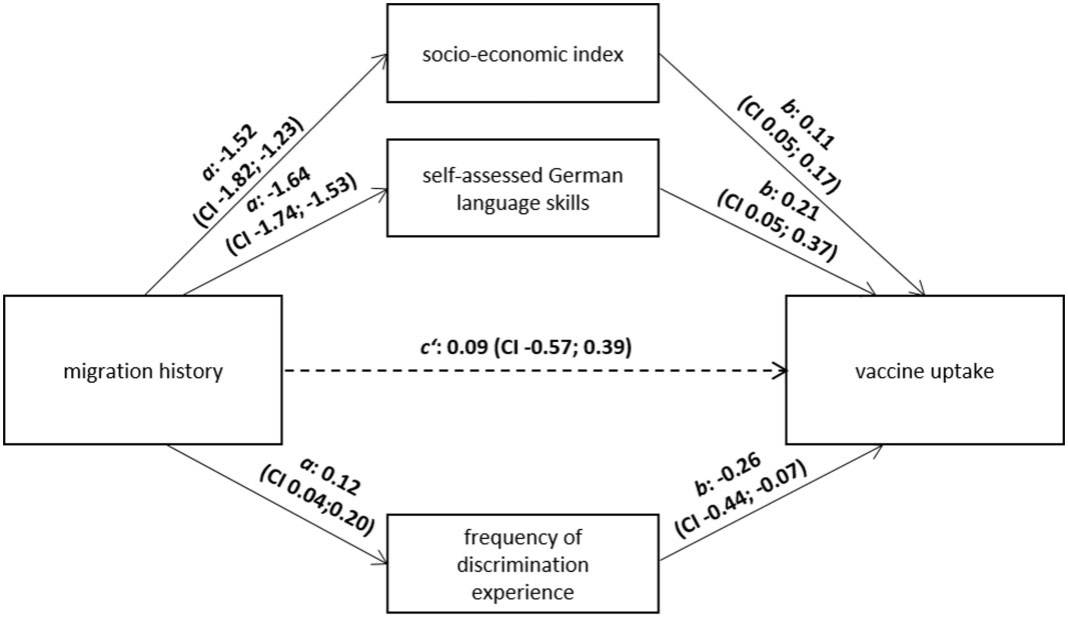
Results of mediation analysis. Note: All coefficients are β coefficients. (Bold denotes significance at *P* < 0.05.) The path coefficient c′ indicates a non-significant direct effect when mediators are included

We further explored whether the socio-economic index, German language skills or discrimination experience mediated the effect of migration history on vaccine uptake (figure 2). The parallel mediation model showed that having a migration history was related to a lower socio-economic index, poorer German language skills and a higher frequency of discrimination experience in the healthcare sector, which in turn were associated with being unvaccinated (indirect effect of socio-economic index: *β *= 0.17, 95% CI: −0.27 to −0.07; indirect effect of German language skills: *β* = −0.34, 95% CI: −0.63 to −0.07; indirect effect of discrimination experience: *β* = −0.03, 95% CI: −0.07 to −0.003). Migration history was not significantly associated with vaccine uptake when the mediator variables were held constant. We, therefore, assumed a complete mediation.

### Psychological determinants of COVID-19 vaccination

We performed two separate multiple logistic regression analyses for participants with and without migration history (table 2). For both study populations, higher confidence, greater conviction that vaccination would bring back freedom and less perceived pressure to get vaccinated increased the likelihood of being vaccinated. For the migrant study population, collective responsibility was positively associated with vaccine uptake. Furthermore, socio-economic index was positively associated with vaccine uptake in the model for the migrant study population. There was no multicollinearity in our models.

**Table 2:** Association between 5C psychological determinants and COVID-19 vaccine uptake: results from logistic regression models

	Migrant sample		Non-migrant sample	
Predictor variables	Odds ratio	(95% CI)	Odds ratio	(95% CI)
(Intercept)	0.06	(0.01–0.54)	0.10	(0.00–20.44)
Confidence	1.42*	(1.03–1.94)	3.67*	(1.82–7.38)
Complacency	0.91	(0.69–1.19)	0.87	(0.47–1.64)
Calculation	1.04	(0.83–1.32)	0.53	(0.24–1.15)
Collective responsibility	1.44*	(1.11–1.86)	1.43	(0.85–2.40)
Constraints	1.02	(0.70–1.49)	1.11	(0.52–2.36)
Pressure	0.62*	(0.49–0.79)	0.60*	(0.39–0.91)
Freedom	1.76*	(1.45–2.13)	1.93*	(1.31–2.85)
Age	1.00	(0.98–1.02)	1.03	(0.99–1.06)
Socio-economic index	1.21*	(1.08–1.35)	1.12	(0.90–1.40)

Observations	681		705	
Mc Fadden’s *R*^2^	0.379		0.690	

* Significance at *P* < 0.05.

## Discussion

One year into the COVID-19 vaccination roll-out, Germany’s population with migration history had, in comparison to the non-migrant population, a significantly lower COVID-19 vaccine uptake but a higher intention to get a first vaccination if they were not already vaccinated. Our data offer some explanation, which point towards inequities due to barriers of language, discrimination and socio-economic status. Psychological determinants further play a role in the decision to vaccinate: higher confidence and higher collective responsibility were associated with higher vaccine uptake.

Our study confirms findings from other surveys —both in Germany and other European countries— that point to lower COVID-19 vaccination coverage among individuals with migration history.^8^^,^^9^^,^^16^ While we found that the vaccination intention of those migrants still unvaccinated was higher than among unvaccinated non-migrants, Crawshaw et al.^16^ found a low intention to vaccinate against COVID-19 among some migrant populations in different European countries. Since intention was high in our study, there must be other reasons for the low vaccination coverage of participants with migration history. These findings are in line with Ohlsen et al.^28^ who found an intention-behaviour gap for COVID-19 vaccination among Spanish-speaking migrants in the USA.

Migration history alone cannot explain vaccine uptake. Other factors associated with migration need to be considered: with higher age, higher socio-economic index, less frequent discrimination experiences, and better German language skills, it is more likely that an individual will be vaccinated. Previous studies showed similar factors, among others, to be associated with routine and COVID-19 vaccine uptake among migrant populations.^16^^,^^29^^,^^30^ There is evidence that people with low socio-economic status^13^ and people who have experienced discrimination in healthcare^11^^,^^12^ use services in the healthcare system less often. This association might also be true for vaccination services and could be one explanation for the observed mediating effect. Furthermore, a language barrier can pose challenges in different steps of the vaccination process.^31^ For example, a language barrier complicates the search for trustworthy information about the vaccine,^16^ which could lead to limited knowledge about the safety and efficacy of the vaccine as well as where to get the vaccine.

Various psychological determinants were associated with COVID-19 vaccine uptake, both in the group with and without migration history. Confidence in the vaccines or the healthcare system plays a major role in the decision to vaccinate all individuals. This finding is in line with other studies where vaccine confidence was a major determinant of vaccine uptake.^19^^,^^32–35^ We also found that individuals with migration history, who see vaccination as a community measure to prevent the spread of COVID-19 (collective responsibility), are more likely to be vaccinated than people who do not. Especially for individuals with a migration history, we expected high perceived constraints to prevent vaccine uptake, but we did not find the expected association. Nevertheless, the strong association between vaccine uptake and German language skills can be interpreted as a specific present barrier.

### Discussion of methods

A particular strength of our survey was the study design and sampling. With our inclusive study design, we tried to overcome language barriers and reach persons, who normally cannot or do not wish to participate in surveys that are offered exclusively in one language. In addition, we considered a strength of our analyses that we developed a DAG for research question Q2 to base our statistical equations on theoretically derived assumptions for causal paths.

COVIMO is a telephone survey that is conducted on behalf of the Robert Koch Institute, the National Public Health Institute in Germany. Both the survey mode and the initiator of the study may result in potential selection biases. Response rates of telephone surveys tend to be lower than those of face-to-face interviews. While the response rate for sample A was 24.2%, no response rate was calculated for sample B. Low response rates increase the risk for a non-response bias, implying that participants may systematically differ from those who chose not to participate, but do not necessarily produce biased results.^36^ Individuals with a more positive attitude towards government authorities and vaccination might have been more likely to participate in this survey. The difference between the estimated one-dose vaccination coverage in our survey sample (90.3%) and the coverage derived from official data (83.9%) supports this assumption.^4^ In addition, our study population had higher education and higher income than the German general population,^10^ and individuals with more than one telephone number were more likely to be included. We addressed these potential biases by weighting the data for education and the quantity of mobile and landscape numbers one participant had, among others. Finally, we cannot exclude socially desirable answers, which are more likely in telephone surveys compared with online surveys.^37^

Different phases of the pandemic with different disease epidemiology as well as different vaccines and vaccination service being available can strongly influence vaccine uptake and vaccination intention as well as psychological determinants. Hence, the transfer of these findings to the future and to other vaccines should only be done with care. Nevertheless, we strongly believe that our findings underline the importance for policy-makers to consider socio-economic status, language and discrimination as potential barriers to vaccination among populations with migration history.

## Conclusion

Our findings indicate a lower uptake of the COVID-19 vaccine in Germany’s population with migration history, compared with the non-migrant population. However, it is not the migrant status, as such, that is the major determinant. Instead, socio-economic status, language and discrimination in the healthcare system should be considered when explaining vaccine uptake and targeting vaccination acceptance.

Reducing barriers through tailored interventions should be one aim of future public health policy to achieve vaccine equity. Building and strengthening confidence in vaccination and healthcare systems as well as spreading the important societal value of vaccination through public health communication can presumably increase vaccine uptake. Actions to achieve this should also be seen as preventative for upcoming vaccine roll-outs to safeguard that no one will be left behind.

## Supplementary Material

ckae017_Supplementary_Data

## Data Availability

The data underlying this article will be shared upon reasonable request to the corresponding author. Key points One year into the COVID-19 vaccine roll-out, Germany’s population with migration history had a lower chance of being vaccinated against COVID-19 but—when still unvaccinated—had a higher intention of getting a first vaccination compared with the non-migrant population. Migration history is not a direct determinant of vaccine uptake; other factors need to be considered: lower socio-economic index, higher frequency of discrimination experienced in the healthcare sector and poorer German language skills are barriers to vaccination. Psychological determinants need to be acknowledged when explaining COVID-19 vaccine uptake: higher confidence and higher collective responsibility increased the chance of individuals with migration history to be vaccinated. Public health policy should reduce barriers through tailored interventions to achieve vaccine equity and should take actions to increase confidence and collective responsibility.
