# Supplementary material for: Barriers and drivers to COVID-19 vaccination among the migrant and non-migrant population in Germany, 2021
Source: Eur J Public Health. 2024 Feb 9;34(3):530–6. doi: 10.1093/eurpub/ckae017 (PMC11161154; doi:10.1093/eurpub/ckae017)
Supplement: ckae017_Supplementary_Data [file ckae017_supplementary_data.pdf]

**Supplementary Material**

Figure S1: Sampling over Time

Figure S2: Directed acyclic graph (DAG) visualizing the association between migration history and vaccine uptake

Table S1: Measures used in survey with sample items and answer formats in the order of their appearance (in the questionnaire)

Available here

Table S2: Association between migration history and COVID-19 vaccine uptake. Results from multiple logistic regression models

**Figure S1.**

*Sampling over time*

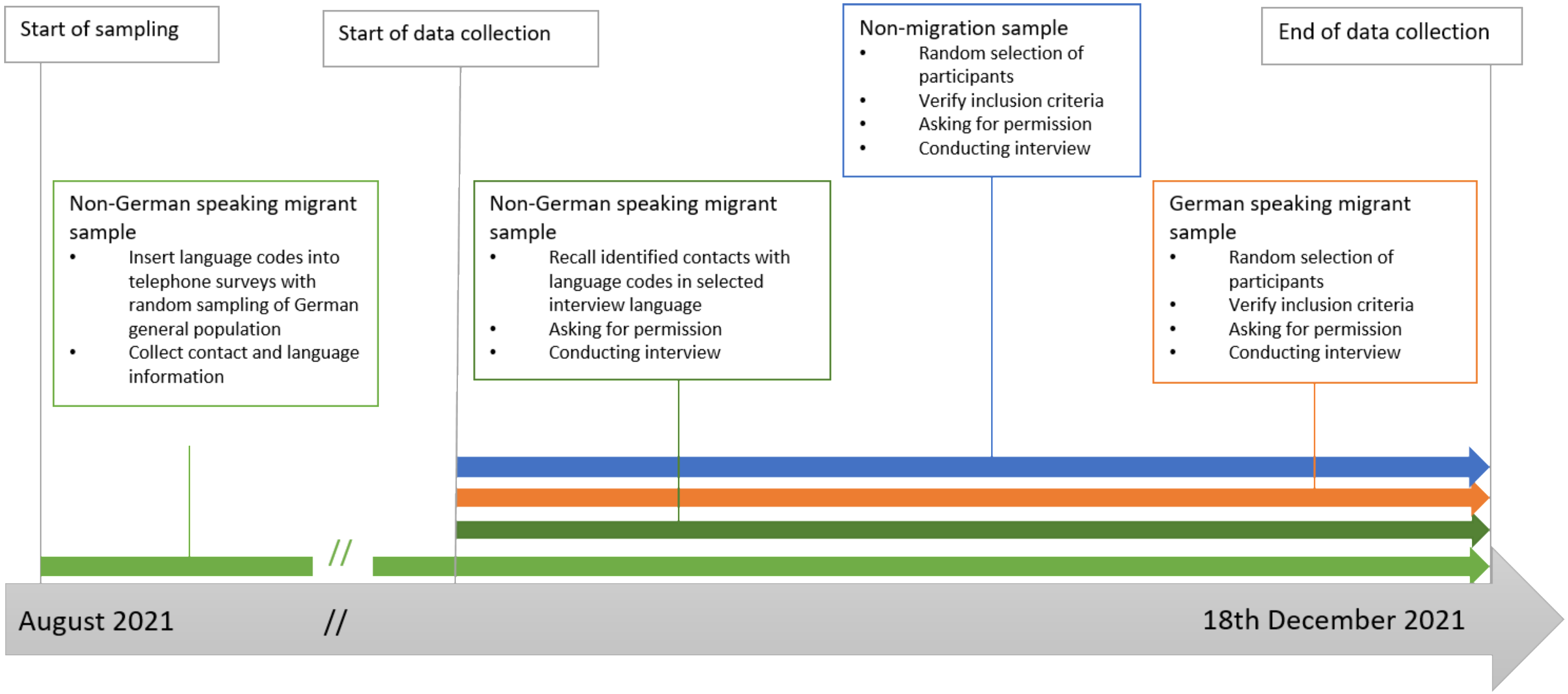

**Figure S2.**

*Directed acyclic graph (DAG) visualizing the association between migration history and vaccine uptake*

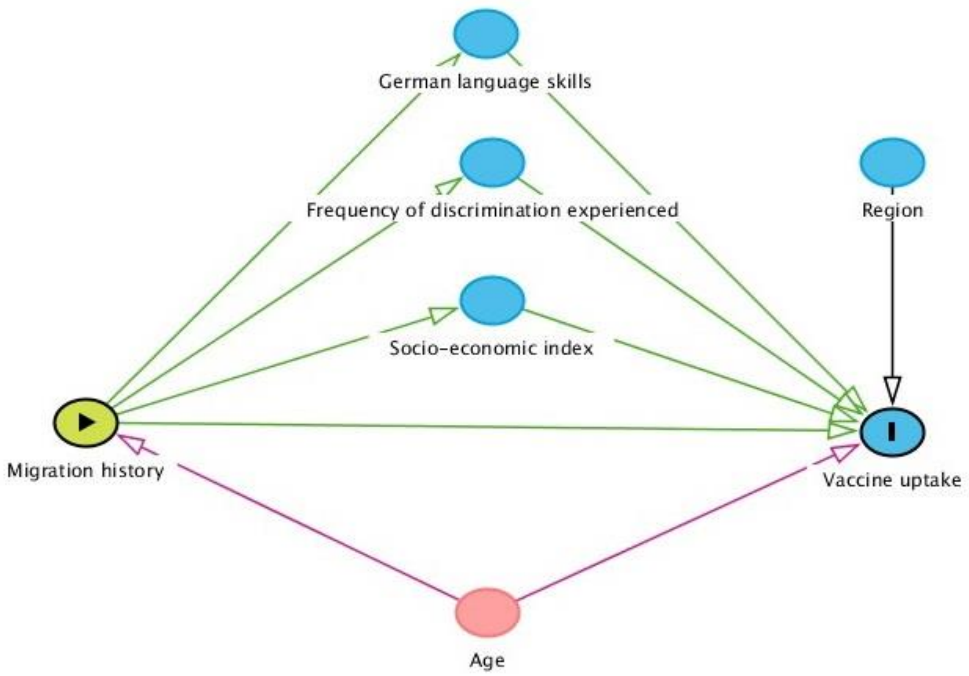

**Table S2.**

*Association between migration history and COVID-19 vaccine uptake. Results from multiple logistic regression models.*

| | Model 1 | | Model 2 | | Model 3 | | Model 4 | | Model 5 (final) | |
|---|---|---|---|---|---|---|---|---|---|---|
| Predictor variable | Odds Ratio | (95 % CI) | Odds Ratio | (95 % CI) | Odds Ratio | (95 % CI) | Odds Ratio | (95 % CI) | Odds Ratio | (95 % CI) |
| (Intercept) | 13.13 | (10.33; 16.68) | 4.84 | (2.95; 7.94) | 1.29 | (0.55; 3.01) | 2.03 | (0.81; 5.10) | 0.64 | (0.17; 2.33) |
| Migration history | | | | | | | | | | |
| Yes | 0.48* | (0.36; 0.65) | 0.50* | (0.37; 0.67) | 0.62* | (0.43; 0.90) | 0.64* | (0.44; 0.93) | 0.92 | (0.57; 1.47) |
| Age | | | 1.02* | (1.01; 1.03) | 1.02* | (1.01; 1.03) | 1.02* | (1.01; 1.03) | 1.02* | (1.01; 1.03) |
| Socio-economic index | | | | | 1.13* | (1.07; 1.20) | 1.14* | (1.07; 1.20) | 1.12* | (1.04; 1.18) |
| Discrimination experience | | | | | | | 0.77* | (0.64; 0.93) | 0.77* | (0.64; 0.93) |
| German language skills | | | | | | | | | 1.23* | (1.05; 1.45) |
| Observations | 2032 | | 2032 | | 1515 | | 1507 | | 1507 | |
| Pseudo $R^2$ | 0.018 | | 0.032 | | 0.048 | | 0.055 | | 0.062 | |

** denotes significance at $p < 0.05$*
